# LAViTSPose: A Lightweight Cascaded Framework for Robust Sitting Posture Recognition via Detection– Segmentation–Classification

**DOI:** 10.3390/e27121196

**Published:** 2025-11-25

**Authors:** Shu Wang, Adriano Tavares, Carlos Lima, Tiago Gomes, Yicong Zhang, Jiyu Zhao, Yanchun Liang

**Affiliations:** 1 School of Computer Science, Zhuhai College of Science and Technology, Zhuhai 519041, China; wangshu11003@zcst.edu.cn (S.W.); 8176156@zcst.edu.cn (Y.Z.);; 2 Department of Industrial Electronics, University of Minho, 4800-058 Guimaraes, Portugal; atavares@dei.uminho.pt (A.T.); clima@dei.uminho.pt (C.L.); mr.gomes@dei.uminho.pt (T.G.)

**Keywords:** sitting posture recognition, semantic segmentation, lightweight Vision Transformer, local consistency regularization

## Abstract

Sitting posture recognition, defined as automatically localizing and categorizing seated human postures, has become essential for large-scale ergonomics assessment and longitudinal health-risk monitoring in classrooms and offices. However, in real-world multi-person scenes, pervasive occlusions and overlaps induce keypoint misalignment, causing global-attention backbones to fail to localize critical local structures. Moreover, annotation scarcity makes small-sample training commonplace, leaving models insufficiently robust to misalignment perturbations and thereby limiting cross-domain generalization. To address these challenges, we propose LAViTSPose, a lightweight cascaded framework for sitting posture recognition. Concretely, a YOLOR-based detector trained with a Range-aware IoU (RaIoU) loss yields tight person crops under partial visibility; ESBody suppresses cross-person leakage and estimates occlusion/head-orientation cues; a compact ViT head (MLiT) with Spatial Displacement Contact (SDC) and a learnable temperature (LT) mechanism performs skeleton-only classification with a local structural-consistency regularizer. From an information-theoretic perspective, our design enhances discriminative feature compactness and reduces structural entropy under occlusion and annotation scarcity. We conducted a systematic evaluation on the USSP dataset, and the results show that LAViTSPose outperforms existing methods on both sitting posture classification and face-orientation recognition while meeting real-time inference requirements.

## 1. Introduction

Sitting posture recognition is a computer vision task that aims to automatically detect, localize, and classify seated human postures, supporting ergonomics assessment and health-risk monitoring in classroom and office settings [1,2,3]. In modern education and workplace environments, prolonged sitting is pervasive; sustained improper posture is linked to abnormal spinal loading and is an independent risk factor for scoliosis, lumbar disc degeneration, and cardiovascular and cerebrovascular diseases [4]. Therefore, scalable posture recognition is critical for early risk screening, ergonomic intervention, and longitudinal evaluation, providing objective measurements beyond sporadic manual assessments [5]. However, in real-world complex scenarios such as classrooms or open offices with multiple people present, frequent occlusions and overlaps between individuals lead to incomplete and ambiguous observations, degrading localization accuracy and complicating fine-grained discrimination. These real-world complexities constitute a key barrier to reliable and scalable posture recognition.

In light of these constraints, early studies predominantly employed wearable sensors or seat-mounted pressure arrays for posture monitoring [6,7,8]. However, limitations in user adherence and scene-level scalability hindered their widespread deployment in classrooms and offices. To reduce intrusiveness and support multi-person coverage, research has gradually shifted toward non-contact, vision-based approaches, among which deep learning has become the core paradigm in recent years due to its consistent performance under occlusion, cross-domain conditions, and fine-grained recognition [9]. For example, DRHN [10] enhances robustness to occlusion by employing hierarchical temporal modeling; SitPose [11] by Jin et al. leverages Kinect for real-time skeletal tracking and joint-angle features, coupled with a soft-voting ensemble to boost accuracy; ASPR [12] integrates multi-scale spatiotemporal skeletal graph convolution with an RNN on local joint angles to fuse spatial, temporal, and whole-body skeletal cues, achieving high-accuracy abnormal sitting posture recognition from the perspective of postural changes. However, the real-world deployment of deep learning models is fundamentally constrained by their dependence on large-scale, high-quality annotations—a resource rendered scarce in real-world settings due to privacy regulations, pervasive occlusions, and prohibitive costs of fine-grained posture labeling. This data scarcity compels the adoption of few-shot learning paradigms, where methods like LGCSPNet [13] leverage structural relationship modeling to maintain spinal misalignment detection reliability with minimal supervision, and JEANIE [14] achieves cross-scenario generalization through temporal–spatial alignment of critical postural transitions both prioritizing diagnostic accuracy for high-risk postures over conventional classification metrics. Yet, in crowded, occlusion-prone environments, relying solely on few-shot methods is insufficient to ensure reliable localization and stable fine-grained discrimination.

Despite these advances, a critical gap persists in real-world multi-person settings: under severe occlusion, limited annotations, and computational constraints, existing vision-based approaches struggle to maintain both spatial fidelity and semantic consistency at the instance level. Specifically, when individuals overlap or are partially visible, detectors often produce imprecise bounding boxes, which in turn corrupt downstream pose parsing and classification. Worse, many deep models—trained with scarce labels—tend to rely heavily on global scene context, inadvertently suppressing discriminative part-level cues (e.g., spine curvature, shoulder alignment) that are essential for distinguishing subtle postural deviations. This leads to two compounding failure modes: (1) error propagation across loosely coupled detection–parsing–classification stages, and (2) degraded robustness to occlusion due to underutilized local structural information. Consequently, even state-of-the-art few-shot methods fail to deliver reliable sitting posture recognition in crowded classrooms or open offices.

From an information-theoretic perspective, these challenges are closely tied to uncertainty and entropy. Occlusion increases uncertainty in the scene, leading to higher entropy in the observations and making the task of detecting and classifying sitting postures more difficult. In particular, when individuals are partially visible or occluded, the mutual information between the input image and the posture labels is reduced, leading to ambiguous or conflicting predictions. Moreover, the scarcity of annotated data compounds this problem, limiting the model’s ability to learn reliable representations and increasing the risk of overfitting to noisy or incomplete labels.

To address these challenges, we propose LAViTSPose, a lightweight cascaded framework for complex multi-person scenes. The pipeline follows a detection–parsing–classification paradigm. In detection, we adopt a YOLOR person detector trained with our proposed Range-aware IoU (RaIoU) loss, which not only yields tight bounding boxes under partial visibility but also helps reduce localization entropy by providing more precise detections in occluded regions, thereby limiting the uncertainty of object detection. In parsing, ESBody, as an entropy filter, with Reno boundary filtering and APF routing, mitigates cross-person leakage and yields part-consistent semantic maps. In classification, a compact Transformer classifier head (MLiT) with Spatial Displacement Contact (SDC) and learnable temperature (LT) strengthens local spatial modeling and stabilizes attention without additional large-scale pretraining beyond standard initialization. While staged for efficiency, the interfaces are explicitly calibrated: segmentation cues serve only for interference suppression and routing, and the classifier consumes skeleton-only inputs, which curbs error propagation between modules. Together, these stages reduce alignment errors and feature contamination from occlusion/overlap and improve sitting posture recognition accuracy and robustness in crowded classrooms/offices under limited annotations, thereby minimizing intra-class entropy and stabilizing attention.

Our contributions can be summarized as follows:

- We present LAViTSPose, a lightweight cascaded framework for complex multi-person indoor scenes. By coordinating occlusion-robust detection, semantic body parsing (ESBody), and a compact Transformer classifier, the framework mitigates the effects of partial visibility and cross-person interference, enabling reliable sitting posture recognition.
- We introduce stage-specific innovations across detection, parsing, and classification. In detection, we propose RAIoU (Range-aware IoU) as the bounding-box regression loss, improving alignment robustness under partial visibility; in parsing, ESBody, with Reno boundary filtering and APF routing, suppresses cross-person leakage and yields part-consistent regions; in classification, a lightweight ViT head combining Spatial Displacement Contact (SDC), learnable temperature, and local structural-consistency regularization strengthens local modeling and robustness under small-sample regimes, without relying on large-scale pretraining.
- We reduce structural uncertainty across the detection–parsing–classification pipeline from an information-theoretic perspective, improving robustness under occlusion and annotation scarcity through entropy-minimizing architectural design.
- We validate the method on the USSP dataset through comprehensive experiments, showing improvements over representative baselines in crowded classroom/office scenarios and strong performance under few-shot settings.

In Section 2, we provide an in-depth review of prior work on sitting posture recognition and related techniques in object detection, semantic segmentation and human parsing, and Transformer-based classification. In Section 3, we provide a detailed introduction to the proposed model, including its workflow, various modules, and technical details. The experimental results and ablation studies are presented in Section 4. Finally, the conclusion and future work are outlined in Section 5.

## 2. Related Work

In this section, we review recent advances most relevant to our method from four perspectives: (1) sitting posture recognition; (2) object detection and bounding-box regression; (3) semantic segmentation and human parsing; and (4) lightweight Transformer-based classification.

### 2.1. Sitting Posture Recognition

Sitting posture recognition aims to automatically determine seated postures to support ergonomic assessment in educational and occupational settings. Early systems largely relied on wearable sensors or seat-embedded pressure sensing: for example, Smart Cushion [15] used seat-cushion FSR arrays for fine-grained recognition; Sensors and Actuators [16] reported a portable pressure-array system combined with machine learning; and Aminosharieh Najafi  [17] presented a multi-sensor smart chair that employed deep learning to classify multiple postures. However, limitations in device cost, user adherence, comfort, maintenance, and scalable deployment have gradually driven research toward non-contact, vision-based paradigms. In this direction, DRHN [10] employs hierarchical temporal modeling to mitigate occlusion and limited visibility, improving robustness on RGB-D sequences; SitPose [11] leverages Kinect for real-time 3D skeletal tracking and joint-angle modeling, coupled with a soft-voting ensemble to improve accuracy; and ASPR [12] utilizes multi-scale spatiotemporal skeletal graph convolution to fuse spatial, temporal, and whole-body structural cues, achieving fine-grained and abnormal sitting posture recognition in cross-instance overlap scenarios.

Existing studies have not, under few-shot constraints, simultaneously achieved occlusion-robust detection for crowded scenes, interference-suppressed segmentation that isolates body parts amid overlaps, and lightweight classification resilient to residual misalignment. However, when detection, parsing, and classification are stitched together without careful interface alignment and error-suppression mechanisms, localization noise can propagate across stages in crowded scenes. Our framework addresses this by equipping each stage with targeted robustness—occlusion-aware detection, interference-suppressed parsing, and structure-regularized classification—so that errors are contained rather than amplified.

### 2.2. Object Detection and Bounding Box Regression

In sitting posture recognition, object detection provides critical “human candidate regions” for subsequent segmentation and classification. The quality of these regions directly determines the discriminability and robustness of downstream features. Current methods fall into two categories:

Region-based detectors prioritize high accuracy but incur significant computational cost. For instance, R-CNN [18] employs Selective Search to generate 2k region proposals, processes each through a CNN for feature extraction, and classifies them using SVMs while refining bounding boxes via linear regression. Cascade R-CNN [19] improves localization by cascading multiple detection heads with progressively higher IoU thresholds. R-FCN [20] enhances efficiency by encoding spatial information into position-sensitive score maps and leveraging position-sensitive region of interest (ROI) pooling, eliminating per-region fully connected layers.

Single-stage detectors emphasize end-to-end dense prediction and real-time performance. YOLO [21] reformulates detection as a unified regression task from image to grid/anchor coordinates, simultaneously predicting objectness, class labels, and bounding boxes. CenterNet [22] adopts an anchor-free paradigm, treating objects as center points on heatmaps and regressing offsets and dimensions, thereby bypassing anchor matching.

Beyond detector architectures, localization quality is improved by IoU-family losses such as GIoU [23] and DIoU/CIoU [24]. These objectives continuously penalize size/shape deviations for all predictions. RIoU [25] further rectifies gradient imbalance by up-weighting high-IoU examples and down-weighting low-IoU ones, thereby emphasizing precise localization; however, it still applies continuous penalties regardless of bbox plausibility. Our RaIoU differs by introducing range-aware intervals on log-width/height/aspect-ratio with near-zero gradients inside the intervals, focusing updates on out-of-range, occlusion-induced outliers typical of classroom/office scenes.

While existing detectors assume proposals cover visible targets, sitting scenarios feature desk/peer occlusions and proximity, causing spatially inaccurate proposals (center misalignment, scale mismatch, partial coverage). This induces critically low IoU, unstable gradients, and error propagation—unaddressed by current methods in modeling occlusion/truncation-induced spatial uncertainty. We thus integrate a lightweight detector with robust regression and quality modeling to deliver precise human regions and stable geometric priors for downstream tasks.

### 2.3. Semantic Segmentation and Human Parsing

Semantic segmentation and human parsing provide pixel-level isolation of individuals in crowded scenes and are commonly used as foundational modules for pose/action recognition. Classic CNN-based approaches such as FCN [26] remain widely adopted for their stability and ease of deployment, particularly in early-stage pipelines. To better model multi-scale context and boundary details in dense environments, subsequent architectures have introduced advanced mechanisms: DeepLabV3+ [27] employs atrous spatial pyramid pooling (ASPP) and encoder–decoder structures to capture global context while preserving fine-grained boundaries; PSPNet [28] leverages pyramid pooling to aggregate multi-region contextual information; HRNet [29] maintains high-resolution representations throughout the network, enabling superior performance on body part delineation tasks.

For real-time applications, lightweight variants such as Fast-SCNN [30] and BiSeNet [31] reduce computational cost through asymmetric encoders or spatial path designs, achieving efficient inference while maintaining reasonable accuracy in multi-person frames. Recent works further integrate semantic segmentation with detection confidence maps or uncertainty estimation—e.g., using adaptive loss weighting [32] or geometric-aware refinement [33]—to mitigate misalignment caused by noisy bounding boxes or occlusions.

Despite these advances, most semantic segmentation methods still treat “person” pixels uniformly without distinguishing individuals, making them vulnerable to cross-person interference when used in detection-based pipelines. Moreover, they rarely incorporate explicit mechanisms to suppress inter-instance contamination or to adapt segmentation boundaries conditioned on detector uncertainty—limitations that we directly address in this work. In particular, Reno suppresses boundary-connected interference from nearby subjects, while APF estimates occlusion and head-orientation cues to route samples, yielding anatomy-aware yet interference-resistant part maps tailored for multi-person seated scenes.

### 2.4. Lightweight Transformer-Based Classification

Transformers, which model long-range dependencies via self-attention, have shown strong performance in image recognition. As the baseline Vision Transformer, ViT [34] partitions an image into non-overlapping patches and performs global self-attention over the resulting tokens. Building on this, Swin Transformer [35] adopts a hierarchical windowing scheme with shifted windows that reduces computation while enhancing local representation capacity, and it has been widely used for dense prediction tasks in addition to classification.

However, in real-world sitting posture recognition, training data are typically limited, costly to annotate, and domain-specific. Under such small-sample conditions, a purely global-attention ViT is more susceptible to background clutter and redundant regions, with particularly unstable attention allocation in multi-person scenes [36]. To improve data efficiency, researchers have explored lightweight/data-efficient Transformers: DeiT [37] introduces a distillation token and supervised distillation from a CNN teacher, enabling data-efficient ViT training using only ImageNet-1k; T2T-ViT [38] performs layer-wise tokens-to-token aggregation to explicitly model local structure and shorten the token sequence; LocalViT [39] injects depthwise separable convolutions into the feed-forward network to strengthen local priors; and LeViT [40] adopts hybrid or mobile-friendly designs to reduce latency and parameter counts while maintaining accuracy. Nevertheless, these approaches largely focus on structural tweaks to the classification backbone, lacking explicit mechanisms for multi-person occlusion and cross-instance interference, and thus struggle to curb attention drift and misclassification at the source. In contrast, our method adopts a cascaded modular design with stage-specific error suppression mechanisms: occlusion-robust detection, interference-aware parsing, and structure-regularized classification. While the pipeline is staged for clarity and efficiency, each module is explicitly designed to isolate and suppress domain-specific errors (e.g., cross-person leakage, partial visibility) that commonly degrade end-to-end learning in crowded scenes. This targeted design—rather than coupling—enables stable training under limited annotations and achieves robust performance in high-interference, small-sample settings.

## 3. Methods

**Figure 1:**
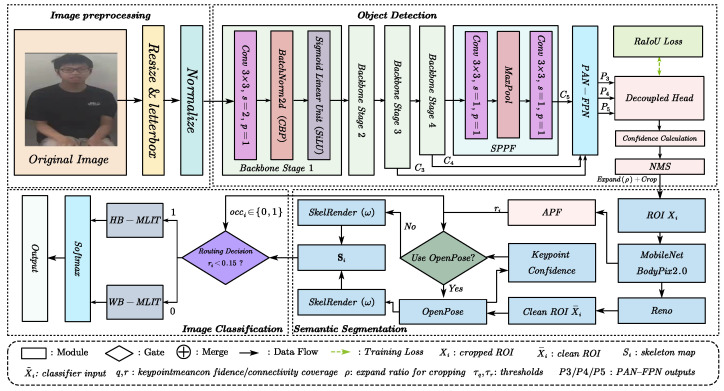
Overview of the LAViTSPose framework. The pipeline begins with object detection to locate all individuals in the input image, retaining only reliable detections and cropping the corresponding regions of interest (ROIs). Semantic segmentation is then applied to each ROI to delineate human body parts, effectively removing interference from other individuals and yielding clean, isolated human silhouettes. This stage also estimates head orientation and determines whether the person is occluded. When feasible, skeletal keypoints are extracted to render a rectangle-based skeleton image that becomes the only input to the classifier; segmentation-derived cues are not concatenated with $S_{i}$ and are used solely for interference suppression and routing. Finally, an image classification module adaptively selects an appropriate processing branch based on the occlusion status and performs pose estimation on the skeleton input, producing the final prediction.

In this section, we first give an overview of our proposed method in Figure 1, which comprises three core modules: (1) object detection, (2) semantic segmentation, and (3) image classification. We then present the key components in detail and finally elaborate on implementation details for both training and inference.

### 3.1. Overview

In multi-person indoor scenes such as classrooms and offices, vision-based seated posture recognition is both practically valuable and technically challenging. The goal is to reliably determine, from a single-frame image, each individual’s posture category (e.g., Upright, Head-on-desk, Leaning-sideways) to support intelligent education, behavior analytics, and human–computer interaction. In real deployments, local occlusions (e.g., arms occluding the torso, front-row students blocking those behind), inter-person overlap (adjacent subjects causing blurred boundaries), and cluttered backgrounds jointly degrade person detection accuracy, contaminate subsequent regional features, and propagate bias into fine-grained classification.

To address these issues, we propose LAViTSPose, a lightweight cascaded framework that structures the pipeline into three specialized stages—detection, segmentation, and classification—each equipped with stage-specific mechanisms to suppress domain-specific errors. In the detection stage, we adopt a YOLOR-based person detector and introduce a Range-aware IoU (RaIoU) loss. RaIoU injects dataset-derived intervals for box width, height, and aspect ratio, keeps gradients near zero for in-range predictions, and applies a saturating penalty only when boxes violate these intervals—while retaining the IoU and a center-alignment term. This range prior improves alignment under severe scale variation and occlusion, yielding tighter localization and less non-target leakage to downstream stages.

In the semantic segmentation stage, we present ESBody. The Remove Non-current Elements (Reno) module employs explicit context suppression to attenuate interference from other individuals, producing clean, single-person masks; the Analysis of Body Part Feature map (APF) module analyzes body part feature maps to infer lower limb occlusion and head orientation. These semantic cues are used only for routing and do not enter the classifier.

In the classification stage, we design a lightweight Vision Transformer variant, MLiT, tailored for posture classification. To compensate for the limited locality of pure attention, MLiT introduces a Spatial Displacement Contact (SDC) operation that injects local inductive bias via pixel-level spatial displacements; meanwhile, a learnable temperature (LT) term is incorporated into attention to dynamically adjust softmax sharpness, stabilizing training and improving generalization.

Overall, LAViTSPose follows a modular path to robust posture recognition under occlusion and overlap: precise person-centric cropping (YOLOR + RaIoU) → interference-resistant parsing with semantic cues (ESBody + Reno/APF) → skeleton-only classification with stabilized attention.

### 3.2. Object Detection

Accurate and tightly fitted human bounding boxes are essential for reliable downstream parsing and classification in multi-person sitting posture recognition. In dense indoor environments—such as classrooms or offices—frequent partial occlusions, significant scale variations, and diverse seated postures often lead conventional detectors to produce loose or misaligned bounding boxes. Such inaccuracies contaminate the regions of interest (ROIs) with neighboring individuals or background clutter, degrading subsequent posture analysis.

To address these challenges, we adopt a YOLOR-based object detector, fine-tuned specifically on indoor seated-scene data to better handle occlusion and scale diversity. Furthermore, we propose a Range-aware IoU (RaIoU) loss that incorporates dataset-derived statistical priors on bounding box width, height, and aspect ratio. This loss suppresses gradient updates for predictions falling within empirically observed valid ranges, while actively penalizing outliers. By doing so, the training process is steered to focus on correcting extreme-scale instances and occlusion-induced misalignments, thereby yielding tighter and more robust detections.

#### 3.2.1. Detector Model

Inspired by the efficient design of the YOLO family [21], we adopt a YOLOR-based [25] person detector, preserving the canonical backbone–neck–head pipeline with task-oriented configurations: SiLU activation, CBP normalization with learnable parameters, PAN multi-scale feature fusion, and anchor re-estimation so that aspect-ratio and scale priors match classroom/office humans. Given an RGB image $x\in R^{H\times W\times 3}$, we preprocess it as

(1) $$x^{\prime }=\mathrm{Preprocess}\left(x\right).$$

The backbone and neck produce a three-level feature pyramid:

(2) $$\left\{C_{3},C_{4}\right\}=\mathrm{Backbone}\left(\tilde{x}\right),\;C_{5}=\mathrm{SPPF}\left(C_{4}\right),\;\left\{P_{3},P_{4},P_{5}\right\}=\mathrm{PAN}\left(C_{3},C_{4},C_{5}\right).$$

On each level $P_{\ell }$ (ℓ∈{3,4,5}), a decoupled head predicts anchor-wise class logits $p^{\left(\ell \right)}$, objectness logits $o^{\left(\ell \right)}$, and bounding-box regressors $b^{\left(\ell \right)}$:

(3) $$\left(p^{\left(\ell \right)},o^{\left(\ell \right)},b^{\left(\ell \right)}\right)=\mathrm{Head}\left(P_{\ell };A_{\ell }\right),$$

where $A_{\ell }$ is the anchor set on level *ℓ*, $p^{\left(\ell \right)}\in R^{|A_{\ell }|\times C}$ denotes the per-anchor class logits for *C* classes, $o^{\left(\ell \right)}\in R^{|A_{\ell }|}$ the per-anchor objectness logits, and $b^{\left(\ell \right)}\in R^{|A_{\ell }|\times 4}$ the anchor-relative box regression outputs. The corresponding decoded boxes are

(4) $$B^{\left(\ell \right)}=\mathrm{decode}\left(b^{\left(\ell \right)},A_{\ell }\right).$$

For each anchor, the candidate confidence score is computed as

(5) $$s^{\left(\ell \right)}=\sigma \left(o^{\left(\ell \right)}\right)⊙\sigma \left(p_{\mathrm{person}}^{\left(\ell \right)}\right),$$

where $p_{\mathrm{person}}^{\left(\ell \right)}\in R^{|A_{\ell }|}$ is the logit corresponding to the *person* class, σ(·) denotes the element-wise sigmoid function, and ⊙ denotes element-wise multiplication.

#### 3.2.2. Range-Aware IoU

Existing IoU-family losses—including continuous size/ratio–augmented variants such as RIoU [25]—typically impose non-zero gradients on all predictions, even when the predicted box width, height, or aspect ratio already falls within empirically reasonable ranges. For instance, RIoU augments IoU with smooth penalties (e.g., L2 loss on $\log(w/w^{*})$), which continuously adjusts all scale deviations regardless of their plausibility. In dense, occluded scenes, this leads to shrink/expand bias, under-emphasis of extreme-scale instances, and instability with truncated boxes.

In contrast, our Range-aware IoU (RaIoU) introduces dataset-derived interval priors for logw, logh, and log(w/h), and employs a piecewise zero-gradient design: predictions within the valid interval incur no penalty, while only out-of-range predictions are corrected via a Huber-type saturating loss. This enables RaIoU to selectively refine anomalous boxes (e.g., severely truncated or misaligned due to occlusion) while preserving well-behaved predictions—thereby reducing unnecessary perturbations and improving robustness. RaIoU can be viewed as introducing a spatial attention mechanism that penalizes high-entropy misalignments in bounding box regression, thus improving downstream parsing certainty. Formally, the total loss is

(6) $$L=\sum_{\ell \in \{3,4,5\}}\left(L_{\mathrm{cls}}^{\left(\ell \right)}+L_{\mathrm{obj}}^{\left(\ell \right)}+\lambda \;L_{\mathrm{RaIoU}}^{\left(\ell \right)}\right).$$

For a predicted box B=(x,y,w,h) and its ground truth $B^{*}=\left(x^{*},y^{*},w^{*},h^{*}\right)$, RaIoU is

(7) $$L_{\mathrm{RaIoU}}=1-\mathrm{IoU}\left(B,B^{*}\right)+\lambda_{\mathrm{pos}}\frac{{\left(x-x^{*}\right)}^{2}+{\left(y-y^{*}\right)}^{2}}{c^{2}+\varepsilon }+\lambda_{r}\;\;\;\;\sum_{z\in \{\log w,\;\log h,\;\log(w/h)\}}\;\;\;\;\phi \;\left(z;\;[a_{z},b_{z}]\right),$$

where *c* is the diagonal of the smallest enclosing rectangle of *B* and $B^{*}$, and ε ensures numerical stability. The interval penalty ϕ(·;[a,b]) is

(8) $$\phi \left(z;\;\left[a,b\right]\right)=\left\{\begin{array}{cc} 0, & a\leq z\leq b,\\ H_{\delta }\left(z-a\right), & z<a,\\ H_{\delta }\left(z-b\right), & z>b, \end{array}\right.\;H_{\delta }\left(t\right)=\left\{\begin{array}{cc} \frac{t^{2}}{2\delta }, & |t|\leq \delta ,\\ \left|t\right|-\frac{\delta }{2}, & |t|>\delta , \end{array}\right.$$

which acts as a Huber-type saturating term outside the interval and equals zero inside.

The intervals $\left[a_{z},b_{z}\right]$ are estimated once from the training set using robust quantiles of the corresponding ground-truth statistics. Here, $Q_{p}\left(\cdot \right)$ denotes the empirical *p*-th quantile (i.e., the inverse CDF evaluated at probability *p*) computed over the training set. Specifically, we set

(9) $$\left[a_{z},b_{z}\right]=\left[Q_{p_{\ell }}\left(z^{*}\right),Q_{p_{u}}\left(z^{*}\right)\right],\;z^{*}\in \left\{\log w^{*},\log h^{*},\log\left(w^{*}/h^{*}\right)\right\},$$

where $p_{\ell }$ and $p_{u}$ are the lower and upper quantile levels, respectively. The exact choices of $\left(p_{\ell },p_{u}\right)$ and other hyperparameters $\left(\delta ,\lambda_{r},\lambda_{\mathrm{pos}}\right)$ are provided in the implementation details.

During inference, we merge candidates across pyramid levels and apply thresholding and NMS:

(10) $$Y=\mathrm{NMS}\;\left(\;\underset{\ell }{⋃}\left\{\left(B^{\left(\ell \right)},s^{\left(\ell \right)}\right)\right\}\;,\;\tau_{\mathrm{obj}},\;\tau_{\mathrm{nms}}\right),$$

and optionally fuse multi-scale results:

(11) $$\hat{Y}=\mathrm{NMS}\;\left(\;\underset{s\in S}{⋃}Y_{s}\right).$$

Each retained box $B_{i}\in \hat{Y}$ is expanded and cropped:

(12) $$X_{i}=\mathrm{Crop}\;\left(x,\;\mathrm{Expand}(B_{i};\rho )\right),$$

and the ROIs $X_{i}$ are passed to downstream parsing and classification.

### 3.3. Semantic Segmentation

Individuals often sit in close proximity, leading to overlapping bounding boxes and the inclusion of non-target body parts within the ROI. Additionally, occlusions from desks, chairs, or adjacent individuals complicate recognition, especially when lower-body visibility is compromised.

**Figure 2:**
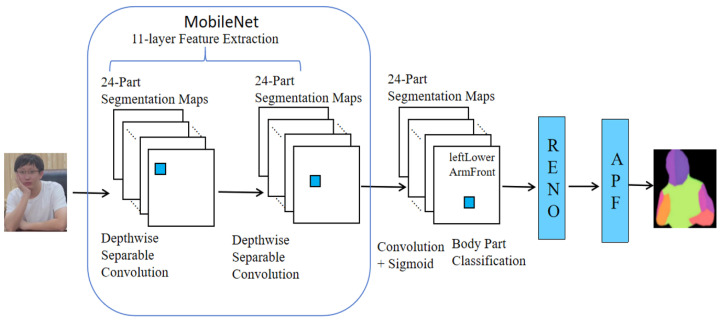
Overall architecture of the proposed ESBody framework, which integrates MobileNet-based 24-part segmentation with RENO and APF modules for interference removal and body part analysis.

As illustrated in Figure 2, we propose ESBody: Efficient and Contextual Semantic Body Parsing, a lightweight, training-free post-processing pipeline built upon the pretrained 24-part MobileNet-BodyPix2.0 [41]. ESBody consists of two modules: Remove Non-current Elements (Reno) and Analysis of Body Part Feature map (APF). Within each detected ROI $X_{i}$, we first produce a binary person mask and retain only the connected component corresponding to the current instance; a thin morphological dilation preserves boundary cues while curbing leakage, yielding a cleaned ROI ${\bar{X}}_{i}$. From ${\bar{X}}_{i}$, APF converts part probabilities into semantic cues for downstream routing.

#### 3.3.1. Remove Non-Current Elements

Reno eliminates interference by suppressing boundary-connected foreground components that likely belong to neighboring persons, as detailed in Algorithm 1. Let $M_{i}^{\left(0\right)}\;\in \;{\left[0,1\right]}^{H^{\prime }\times W^{\prime }}$ be the BodyPix foreground probability within $X_{i}$; we binarize and then apply boundary-driven filtering and a thin dilation:

(13) $${\tilde{M}}_{i}=1\left[M_{i}^{\left(0\right)}\geq \tau_{m}\right],\;{\hat{M}}_{i}=\mathrm{BoundaryFilter}+\mathrm{Dilate}\left({\tilde{M}}_{i};\;T,\kappa \right),\;{\bar{X}}_{i}={\hat{M}}_{i}⊙X_{i},$$

where $\tau_{m}$ is the binarization threshold, $T=\tau_{\ell }\left(m+n\right)$ is the length threshold for boundary-connected components (default $\tau_{\ell }=1.0$), κ is the dilation radius, and ⊙ denotes element-wise masking.
**Algorithm 1:** Reno: Boundary-connected component suppression.
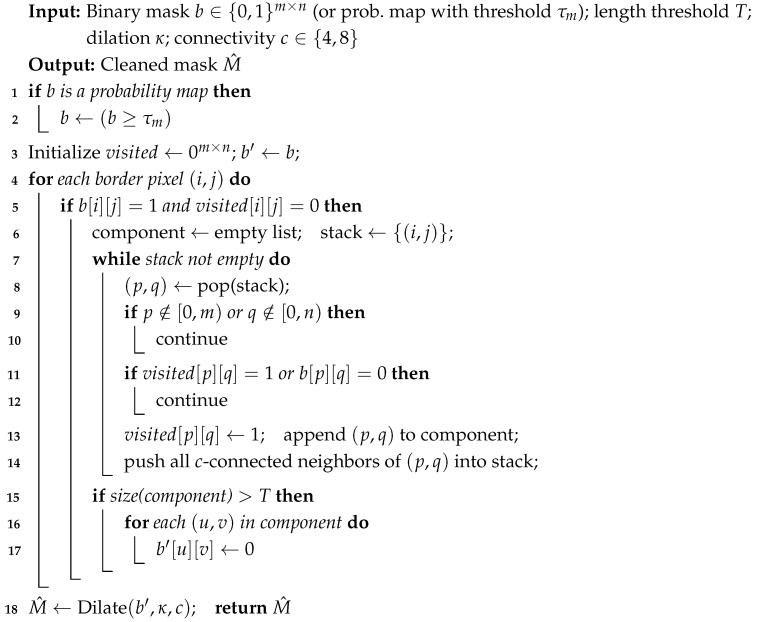


If the confidence-weighted torso centroid $c_{i}$ lies inside the foreground, we first retain the connected component containing $c_{i}$ before boundary suppression.

#### 3.3.2. Analysis of Body Part Feature Map

APF converts BodyPix’s soft part probabilities on ${\bar{X}}_{i}$ into two semantic cues and a routing decision, without any training. Let $\Pi_{i}=\psi \left({\bar{X}}_{i}\right)\in {\left[0,1\right]}^{h\times w\times K}$ be the fixed BodyPix output and

(14) $$A_{i,k}\left(u\right)=\frac{\Pi_{i}\left(u,k\right)}{\sum_{t=1}^{K}\Pi_{i}\left(u,t\right)+\varepsilon },$$

where K=24, *u* indexes the h×w grid, and ε ensures numerical stability.

Lower-body visibility ratio. With $P_{\mathrm{lb}}$ the index set of lower-body parts (hips, thighs, calves, feet),

(15) $$r_{i}=\frac{\sum_{u}\sum_{k\in P_{\mathrm{lb}}}A_{i,k}\left(u\right)}{\sum_{u}\sum_{k=1}^{K}A_{i,k}\left(u\right)}.$$

Facial orientation inference. Using left/right facial regions, we derive a coarse orientation by comparing their activations:

(16) $${\mathrm{pos}}_{i}=\left\{\begin{array}{cc} -1, & d_{i}\left[\mathrm{leftFace}\right]>1.5\;d_{i}\left[\mathrm{rightFace}\right],\\ \;\;0, & 0.67\;d_{i}\left[\mathrm{leftFace}\right]\leq d_{i}\left[\mathrm{rightFace}\right]\leq 1.5\;d_{i}\left[\mathrm{leftFace}\right],\\ \;\;1, & d_{i}\left[\mathrm{rightFace}\right]>1.5\;d_{i}\left[\mathrm{leftFace}\right], \end{array}\right.$$

where $d_{i}\left[\cdot \right]$ is the spatial average of the corresponding part attention.

Occlusion-aware routing (APF-only). We declare lower-body occlusion if $r_{i}<0.15$ and choose the branch

$$b\left(i\right)=\left\{\begin{array}{cc} \mathrm{HB}, & r_{i}<0.15,\\ \mathrm{WB}, & r_{i}\geq 0.15. \end{array}\right.$$

APF cues $\left(r_{i},{\mathrm{pos}}_{i}\right)$ are used only for routing and are not concatenated with the classifier input. The branch-specific prediction is

$${\hat{y}}_{i}=f_{b(i)}\left(S_{i}\right).$$

where $f_{\mathrm{HB}}$ and $f_{\mathrm{WB}}$ are two posture classifiers specialized for half-/upper-body and whole-body evidence, respectively. APF itself contains no learnable parameters; it operates entirely on BodyPix outputs using geometric heuristics.

The APF module enriches raw segmentation maps with high-level semantic understanding by analyzing anatomical pixel distributions, inferring occlusion status from lower-body visibility, and estimating facial orientation through asymmetric facial features. These semantic cues enhance downstream feature representation and improve robustness for posture recognition in occlusion-prone environments, without requiring any training.

#### 3.3.3. Human Pose Estimation with OpenPose

**Figure 3:**
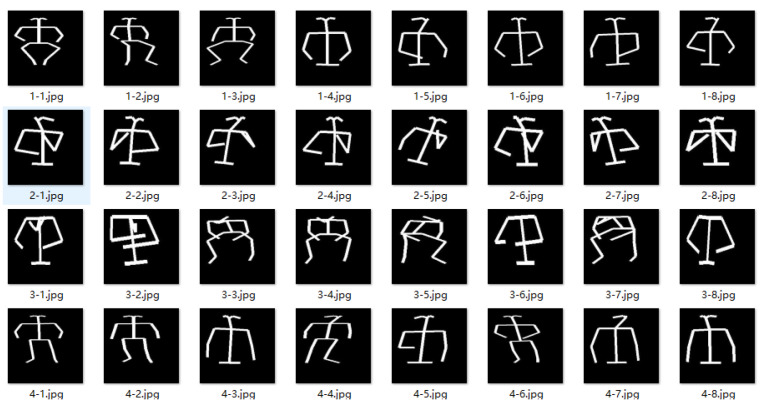
Examples of the proposed rectangle-based binary skeleton. Unlike thin-line renderings, limbs are solid rectangles (default width ω = 4) to enhance structural continuity and feature density, improving robustness to keypoint noise.

To address residual ambiguities, we introduce OpenPose as an inference-only geometric prior and skeleton generator: (i) the confidence-weighted torso centroid provides a robust reference point $c_{i}$ for Reno; and (ii) the rendered rectangle-based skeleton $S_{i}$ is the only input to the classifier. Examples of the generated binary skeletons are shown in Figure 3, as illustrated below. When the aggregate torso-keypoint confidence is low $\left(\sum_{j\in J_{\mathrm{torso}}}\kappa_{i}^{j}<\tau_{\mathrm{pose}}\right)$, we fall back to the detection-box center for $c_{i}$. The pose estimator is frozen and used only at inference; no gradients are backpropagated.

We define the confidence-weighted torso centroid as

$$c_{i}=\frac{\sum_{j\in J_{\mathrm{torso}}}\kappa_{i}^{j}\;p_{i}^{j}}{\sum_{j\in J_{\mathrm{torso}}}\kappa_{i}^{j}},\;p_{i}^{j}=\left(x_{j},y_{j}\right).$$

For rendering, we use a rectangle-based skeleton. For each person, we form a tight keypoint box from confident joints $J_{\mathrm{eff}}=\left\{j\;|\;\kappa_{i}^{j}\geq \tau_{\mathrm{kp}}\right\}$ (default $\tau_{\mathrm{kp}}$ = 0.3). If $|J_{\mathrm{eff}}|\geq 2$, define

(17) $$x_{\min}=\min_{j\in J_{\mathrm{eff}}}x_{j},\;x_{\max}=\max_{j\in J_{\mathrm{eff}}}x_{j},\;y_{\min}=\min_{j\in J_{\mathrm{eff}}}y_{j},\;y_{\max}=\max_{j\in J_{\mathrm{eff}}}y_{j},$$

otherwise fall back to the detection box (or ${\bar{X}}_{i}$). We map keypoints to a 224 × 224 canvas using aspect-preserving letterbox scaling:

(18) $$\begin{array}{cc} \; & w=x_{\max}-x_{\min},\;h=y_{\max}-y_{\min},\;s=\frac{224}{\max(w,h)},\\ \; & \delta_{x}=\frac{1}{2}\left(224-s\;w\right),\;\delta_{y}=\frac{1}{2}\left(224-s\;h\right),\\ \; & x^{\prime }=s\;\left(x-x_{\min}\right)+\delta_{x},\;y^{\prime }=s\;\left(y-y_{\min}\right)+\delta_{y}. \end{array}$$

For a limb between $\left(x_{1}^{\prime },y_{1}^{\prime }\right)$ and $\left(x_{2}^{\prime },y_{2}^{\prime }\right)$, the orientation is

(19) $$\theta =atan2(y_{2}^{\prime }-y_{1}^{\prime },\;x_{2}^{\prime }-x_{1}^{\prime }),$$

and the half-width offsets along the perpendicular direction are

(20) $$\Delta x=-\sin\left(\theta \right)\cdot \frac{\omega }{2},\;\Delta y=\;\cos\left(\theta \right)\cdot \frac{\omega }{2}.$$

**Figure 4:**
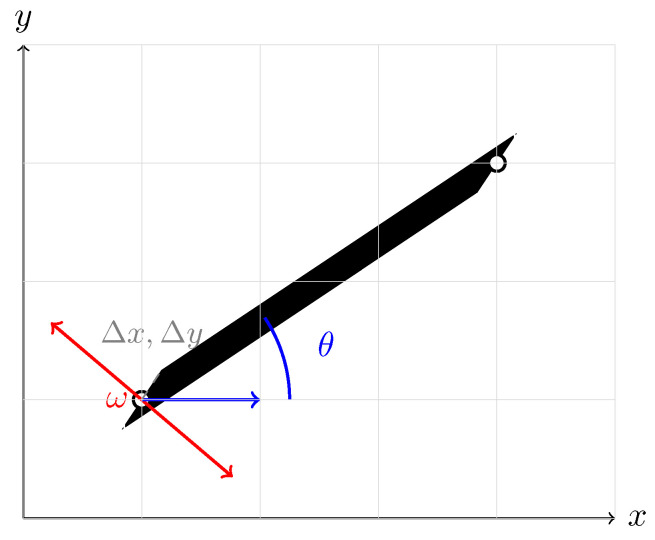
Illustration of the rectangle-based skeleton rendering. Two adjacent keypoints are connected by a solid rectangle of width ω.

The four vertices are

(21) $$\begin{array}{ccc} v_{1} & =\left(x_{1}^{\prime }+\Delta x,\;y_{1}^{\prime }+\Delta y\right),\;v_{2} & =(x_{1}^{\prime }-\Delta x,\;y_{1}^{\prime }-\Delta y),\\ v_{3} & =\left(x_{2}^{\prime }-\Delta x,\;y_{2}^{\prime }-\Delta y\right),\;v_{4} & =(x_{2}^{\prime }+\Delta x,\;y_{2}^{\prime }+\Delta y), \end{array}$$

which are rasterized and filled to obtain a limb mask. The geometric construction is illustrated in Figure 4, as shown below. Let E denote the standard COCO-18 limb set. We build a binary skeleton image $S_{i}$$\in {\left\{0,1\right\}}^{224\times 224}$ by filling rectangles for all e∈E whose endpoints are present in $J_{\mathrm{eff}}$; limb strength can be modulated by $w_{e}=\min\left(\kappa_{i}^{a},\kappa_{i}^{b}\right)$ if a grayscale skeleton is desired.

Segmentation remains necessary: Reno reduces cross-person leakage inside the ROI, preventing erroneous keypoint grouping and stabilizing skeletal topology; APF provides lower-body occlusion and coarse head orientation to route samples into HB/WB branches. These cues improve the reliability of the skeleton representation and the choice of the appropriate classifier, without being concatenated with $S_{i}$.

### 3.4. Image Classification

**Figure 5:**
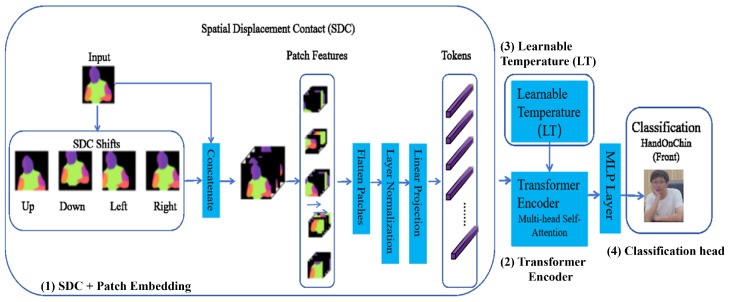
Overview of the MLiT architecture. The numbered blocks in the diagram correspond to the following: (1) SDC-enhanced patch embedding, where the input image is first augmented by directional spatial shifts and then split, flattened, and linearly projected into token embeddings; (2) Transformer encoder, a stack of Transformer blocks with multi-head self-attention processing the figure token sequence, with a prepended [CLS] token for classification; (3) LT module, where a learnable temperature τ controls the sharpness of the softmax distribution in the attention computation; (4) classification head, which applies a lightweight MLP to the final [CLS] token to predict the sitting posture category.

The final stage of LAViTSPose is an image classification module built on a standard Vision Transformer (ViT). Although ViT leverages global self-attention to capture long-range dependencies, it lacks inductive bias toward local spatial structure. To overcome this limitation, we propose MLiT (Modular Lightweight Image Transformer), a lightweight and efficient Transformer-based classifier tailored for small-scale, occlusion-prone datasets. As shown in Figure 5, MLiT introduces two key innovations: (1) a Spatial Displacement Contact (SDC) module in the patch-embedding stage to enhance local spatial awareness; and (2) a learnable temperature (LT) mechanism to stabilize the attention distribution during training. With these designs, MLiT maintains high classification accuracy while using substantially fewer parameters, demonstrating effectiveness on small datasets commonly encountered in specialized tasks such as sitting posture recognition.

#### 3.4.1. Spatial Displacement Contact

Standard ViTs tokenize an image by non-overlapping patch projection, discarding fine local structures. SDC injects local inductive bias by aggregating a few spatially displaced neighbors before patchifying. Given $x\in R^{H\times W\times C}$ and Δ={(0,0),(±δ,0),(0,±δ),(±δ,±δ)}, define a shift operator $S_{d}\left(\cdot \right)$ (reflection padding) and form a concatenated feature map:

(22) $$\tilde{x}={\mathrm{Concat}}_{d\in \Delta }\;\left[S_{d}\left(x\right)\right]\in R^{H\times W\times (C|\Delta |)}.$$

Tokens are obtained by unfolding $\tilde{x}$ into P×P patches and a linear projection:

(23) $$T=\mathrm{Unfold}\left(\tilde{x};P\right)\in R^{N\times (P^{2}C|\Delta |)},\;z_{0}=TW_{e}+b\in R^{N\times D},$$

where $N=\frac{HW}{P^{2}}$, *D* is the embedding dimension, and $W_{e}\in R^{(P^{2}C|\Delta |)\times D}$. We set δ = 1 pixel and use the 4-neighborhood by default; the 8-neighborhood is optional. A difference-augmented variant is

(24) $$\hat{x}=\mathrm{Concat}\;\left[x,\;{\left\{S_{d}\left(x\right)-x\right\}}_{d\in \Delta \setminus \{(0,0)\}}\right],$$

which emphasizes local intensity changes useful under occlusion.

In the context of posture recognition, SDC can be viewed as implicitly minimizing intra-class entropy in the feature space. By aggregating local spatial context, SDC enhances the model’s ability to preserve discriminative part-level features, which are crucial for distinguishing subtle postural variations, even under occlusion or annotation noise. This structural-consistency regularization encourages features to cluster tightly within the same postural class, thus improving robustness to occlusion and reducing the uncertainty that arises in information-scarce settings. Consequently, the incorporation of SDC aids in mitigating entropy-related uncertainty in the model’s predictions, thereby enhancing the reliability of posture recognition without the need for large-scale supervision.

#### 3.4.2. Learnable Temperature

Training Transformers on small datasets often produce unstable attention patterns. We therefore introduce a learnable temperature (LT): instead of a fixed temperature, τ is a trainable scalar optimized jointly with the network,

(25) $${\mathrm{softmax}}_{\tau }\left(a_{i}\right)=\frac{\exp(a_{i}/\tau )}{\sum_{j}\exp\left(a_{j}/\tau \right)},$$

allowing the model to adapt attention entropy during training.

#### 3.4.3. Classification Head and Training Objective

Each detected person yields a rectangle-based skeleton image $S_{i}$ from OpenPose (inference-only). APF provides routing variables $\left(r_{i},{\mathrm{pos}}_{i}\right)$ but these cues are not fed to the classifier. According to $r_{i}$, the sample is routed to the HB/WB branch. Let $I_{i}=S_{i}$ be the classifier input and $z_{0}$ the token matrix after SDC patch embedding (with positional embeddings and a class token). We stack *L* pre-norm Transformer blocks with learnable temperature τ:

(26) $$\begin{array}{cc} U_{\ell } & =Z_{\ell -1}+{\mathrm{MSA}}_{\tau }\;\left(\mathrm{LN}(Z_{\ell -1})\right),\\ Z_{\ell } & =U_{\ell }+\mathrm{MLP}\;\left(\mathrm{LN}(U_{\ell })\right),\;\ell =1,\ldots ,L, \end{array}$$

and take the [CLS] token representation

(27) $$h_{i}=\mathrm{LN}\;\left(Z_{L}^{\left[\mathrm{CLS}\right]}\right).$$

Logits and probabilities are

(28) $${\hat{y}}_{i}=W_{o}h_{i}+b_{o},\;p_{i}=\mathrm{softmax}\left({\hat{y}}_{i}\right),$$

where $W_{o}\;\in \;R^{C\times D}$, $b_{o}\;\in \;R^{C}$, and *C* is the number of posture classes.

With APF routing (Section 3.3.2), each sample is assigned to a branch b(i)∈{HB,WB} according to $r_{i}$. Let $p_{i}^{\mathrm{HB}}$/$p_{i}^{\mathrm{WB}}$ be the predicted distributions from the corresponding MLiT head (same architecture, separate parameters). The training objective is

(29) $$L_{\mathrm{post}}=\sum_{i\in S_{\mathrm{HB}}}\mathrm{CE}\;\left(y_{i},\;p_{i}^{\mathrm{HB}}\right)+\sum_{i\in S_{\mathrm{WB}}}\mathrm{CE}\;\left(y_{i},\;p_{i}^{\mathrm{WB}}\right),$$

where $S_{\mathrm{HB}}=\left\{i\;|\;r_{i}<\tau_{\mathrm{lb}}\right\}$ and $S_{\mathrm{WB}}=\left\{i\;|\;r_{i}\geq \tau_{\mathrm{lb}}\right\}$. At inference, we output $\arg\max_{k}p_{i,k}^{\;b(i)}$.

In summary, the framework is designed as a progressive pipeline: YOLOR ensures precise person localization, ESBody removes cross-person interference and identifies occlusion, and OpenPose converts the refined regions into structured skeletons that are finally classified by MLiT. Each module builds upon the previous one, forming a coherent system for robust posture recognition in crowded environments.

## 4. Experiments

In this section, we conduct extensive experiments on the sitting posture recognition task. We then qualitatively and quantitatively analyze the advantages of our proposed method compared with existing approaches. Furthermore, we evaluate the effectiveness of each key component through ablation studies.

### 4.1. Datasets

In this work, we employ the University Student Sitting Posture (USSP) dataset [42], a few-shot real-world benchmark specifically curated for fine-grained sitting posture and head-orientation recognition in multi-person scenes. Collected across diverse indoor environments—including classrooms, dormitories, study rooms, and offices—images typically contain multiple individuals seated in close proximity, with frequent occlusions from desks, chairs, and neighboring people. The dataset contains 2952 annotated images, split 8:2 into 2362 training and 590 test samples. Each visible seated individual is annotated at the instance level (person bounding box and categorical labels for sitting posture and head orientation). All annotations were independently produced by multiple annotators and subsequently validated for consistency, with high inter-annotator agreement. Although compact, USSP spans diverse scenarios and posture variations under occlusion and overlap, enabling robust evaluation of behavior-recognition models in realistic, low-resource, multi-person conditions.

### 4.2. Implementation Details

We implement LAViTSPose in PyTorch 1.12.1 on an NVIDIA RTX 4090 GPU. Input skeleton maps are generated via OpenPose by extracting 18 body keypoints per subject, rendering them into binary skeleton images, filtering for quality, and resizing to 224×224. The classifier jointly predicts sitting posture and head orientation. During training, we use the Adam optimizer with an initial learning rate of $1\times 10^{-5}$ and a batch size of 16; the schedule adopts a 10% warm-up followed by linear decay.

Only the detector and the classifier are trainable. The detector is optimized with RaIoU; ESBody is a training-free parsing module built on BodyPix (frozen), and OpenPose is used inference-only with no gradients backpropagated. The classifier (MLiT) is trained on skeleton-only inputs $S_{i}$; ESBody-derived cues (occlusion and head orientation) are used solely for routing and are not concatenated with $S_{i}$. For detector-sweep and loss ablations, ESBody and MLiT are trained once using our YOLOR-based detector and then kept frozen while we retrain only the detector under the same protocol. Unless otherwise noted, all FPS are measured on the same RTX 4090 in PyTorch eager mode (TensorRT disabled) with single-image inference; detector and classifier use their respective default input resolutions.

### 4.3. Evaluation Metrics

We evaluate LAViTSPose using standard classification metrics: accuracy, precision (P), recall (R), and F1-score. Accuracy reflects the overall correctness of predictions across all classes, serving as a general performance indicator. Precision measures the proportion of correctly predicted positives among all instances classified as positive, indicating how “trustworthy” the positive predictions are. Recall quantifies the model’s ability to identify all actual positives, revealing its sensitivity to minority or easily missed classes. The F1-score, as the harmonic mean of precision and recall, provides a balanced measure particularly valuable under class imbalance a common challenge in posture and orientation datasets where certain poses or directions occur less frequently. To further assess computational efficiency, we report the average inference time per image (in milliseconds). All metrics are computed separately for the sitting posture and facial-orientation tasks, and results are presented as macro-averages across classes to ensure equal weighting regardless of class size—thus offering a fair evaluation under imbalanced label distributions.

### 4.4. Comparison with State of the Art

We conduct a comprehensive comparison between our proposed LAViTSPose and a diverse set of state-of-the-art architectures across multiple dimensions: accuracy, efficiency, model complexity, and inference latency. As summarized in Table 1, LAViTSPose achieves a new state-of-the-art accuracy of 94.23% on the sitting posture recognition task, surpassing all state-of-the-art (SOTA) models by a clear margin. More importantly, it strikes an exceptional balance among performance and practicality—achieving this high accuracy with only 54.2M parameters and an inference time of 34.17 ms per sample, making it highly suitable for real-time deployment in edge-constrained environments.

LAViTSPose also delivers 92.02% precision and 92.22% F1, reflecting strong classification confidence and balanced cross-class performance. Although its recall (92.34%) is slightly lower than ViT (94.58%), the gains in computational efficiency and aggregate metrics offset this minor gap. ViT benefits from strong pretraining priors and global attention but at substantially higher cost—85.7M parameters, 16.9 GFLOPs, and 36.13 ms per image—i.e., roughly +58% parameters and FLOPs relative to LAViTSPose with slower inference. In resource-sensitive settings, such marginal recall gains come at a disproportionate coputational expense.

By contrast, lightweight model MobileNet excels in efficiency (2.23M parameters; 0.33 GFLOPs) but exhibits pronounced performance drops: 87.76% accuracy, with concomitant declines in precision and F1-score. This gap underscores the difficulty of preserving sufficient representational capacity under extreme parameter compression, particularly for fine-grained posture discrimination that demands strong spatiotemporal sensitivity.

Notably, advanced Transformer variants PiT and CaiT introduce hierarchical or progressive attention, yet do not surpass ViT; despite sizable models (up to 121.3M parameters), their accuracy remains around 90.8%. Without task-specific inductive biases, added architectural complexity yields diminishing returns—whereas LAViTSPose narrows this gap through a task-tailored design aligned with the demands of sitting posture recognition.

Overall, the results substantiate the effectiveness of the proposed framework on both sitting posture and facial-orientation recognition. Ablation studies further verify the necessity of each component, with the APF module contributing the largest gains. Decomposing the task into specialized, interdependent stages—detection, segmentation, and classification—proves highly effective, and the combination of high accuracy with reasonable computational cost makes LAViTSPose a practical solution for real-world monitoring systems.

### 4.5. Ablation Studies

To validate the contribution of each component in the LAViTSPose framework, we conduct a series of ablation studies.

#### 4.5.1. Ablation Analysis of Object Detection Key Components

The ablation study on object detection components reveals the critical importance of each architectural enhancement in addressing the unique challenges of human detection in multi-person sitting scenarios. As shown in Table 2, the baseline model (without specialized detection components) achieves only 81.12% accuracy, highlighting the severe limitations of standard detectors when confronted with the pervasive occlusions and scale variations characteristic of classroom and office environments.

The CBP normalization layer alone (Settings (a)) provides a 2.12 percentage point improvement in accuracy to 83.24%, confirming its effectiveness in adapting to the diverse input statistics encountered in real-world settings. This aligns with the module’s design principle of handling varying input statistics through learnable parameters, which is particularly valuable in multi-person scenes where lighting conditions, camera angles, and person sizes vary significantly. In occlusion-prone environments, stable feature normalization becomes crucial for consistent objectness scoring across different spatial contexts.

The addition of SiLU activation (Settings (b)) further boosts accuracy to 85.38%, revealing the importance of non-linear activation properties for discriminative feature learning. Unlike traditional activation functions, SiLU’s smooth, non-monotonic nature provides enhanced representation capacity for modeling subtle boundary distinctions between adjacent individuals. This improvement is particularly significant in crowded settings where small localization errors can cause non-target leakage into subsequent processing stages.

The most substantial gain comes from implementing PAN-FPN (Settings (c)), which increases accuracy by 5.21% to 86.33%. This dramatic improvement validates PAN-FPN’s ability to effectively fuse multi-scale features, addressing the critical challenge of severe scale variations in multi-person scenes. In classroom and office settings, individuals can appear at vastly different scales depending on their distance from the camera and seating position, making multi-scale integration essential for consistent detection performance. The 4.89 percentage point increase in F1 score (from 82.01% to 86.90%) underscores PAN-FPN’s particular value in maintaining high precision while capturing small or partially occluded individuals.

The synergistic effect of all three components in the complete LAViTSPose system is striking: it achieves 94.23% accuracy, a 7.90 percentage point improvement over the best single-component configuration and a 13.11 percentage point gain over the baseline. This non-additive gain reveals a critical interdependence between these modules. CBP provides stable feature normalization across varying conditions; SiLU enhances the discriminative capacity for boundary definition; and PAN-FPN ensures robustness across scale variations. Together, they create a detection system that can reliably isolate individuals even under severe occlusion, providing the foundation for the entire pipeline.

#### 4.5.2. Ablation Study on YOLO Architecture Variants

This comparative evaluation reveals the strategic advantage of our customized YOLOR variant within the LAViTSPose framework. To ensure a fair comparison, all detectors—including YOLO-v3 through YOLO-v11, the original YOLOR [25], and our customized YOLOR—are trained independently from scratch on the same data with identical input resolution, augmentations, and training protocol. Crucially, the downstream pipeline is held fixed: the ESBody segmentation module and MLiT classifier are trained once using ROIs from our customized YOLOR and then frozen across all detector variants. No retraining or fine-tuning is performed when swapping detectors.

As shown in Table 3, our customized YOLOR achieves the highest end-to-end classification accuracy (94.23%) and F1-score (92.18%), outperforming the strongest baseline, YOLO-v11 (94.03% Acc, 92.17% F1). Notably, while YOLO-v11 attains slightly higher precision (92.20% vs. 92.02%), our method achieves superior recall (92.34% vs. 92.14%), resulting in a better-balanced F1 score. This gain is directly supported by superior detection performance: as reported in Table 4, our method attains the highest mAP@0.5 of 93.12%, surpassing YOLO-v11 (92.60%) and the original YOLOR (91.24%). The inference speed of our detector (60.15 FPS) remains competitive with YOLO-v11 (65.37 FPS) and faster than most YOLO variants.

The consistent improvement over both generic YOLO architectures and the original YOLOR stems from task-specific enhancements: SiLU activation (vs. Mish in YOLOR), CBP normalization, PAN-FPN neck, anchor re-estimation, and our RaIoU loss. These refinements collectively address the unique challenges of seated-posture scenes—partial occlusion, scale variation, and proximity-induced overlap. Although the absolute accuracy gain over YOLO-v11 is modest (0.20%), it represents a 3.35% relative error reduction (from 5.97% to 5.77%). In ergonomic monitoring, where each misclassification may indicate an undetected health risk, this improvement is both statistically significant and operationally meaningful.

#### 4.5.3. Ablation Study on Bounding Box Regression Losses

The ablation study on bounding box regression losses is conducted under a strictly controlled setting: all loss variants (IoU, DIoU, CIoU, GIoU, RIoU, and RaIoU) are evaluated on the same YOLOR detector, with identical backbone, neck, head architecture, data augmentation, optimizer, learning rate schedule, and hyperparameters—only the regression loss term is varied. As shown in Table 5, RaIoU significantly outperforms conventional loss functions, achieving 94.23% accuracy compared to 88.42–90.21% for baseline methods. This substantial 4.02–5.81 percentage point improvement demonstrates the critical importance of specialized regression mechanisms for occlusion-prone scenarios.

The performance gap becomes particularly pronounced when examining precision and F1 scores. RaIoU achieves 92.02% precision and 92.18% F1, indicating its superior ability to minimize false positives while maintaining high true positive rates. This is crucial for downstream tasks, as even small localization errors in crowded classroom or office settings can cause non-target leakage—where adjacent individuals’ body parts contaminate the region of interest, leading to cascading errors in segmentation and classification.

The key insight revealed by these results is that traditional IoU-based losses (including aspect-ratio-aware variants like DIoU [24], CIoU [49], GIoU [23], and RIou [25]) fundamentally fail to model the spatial uncertainty induced by desk/peer occlusions and proximity in sitting scenarios. While these losses consider geometric relationships between bounding boxes, they remain inadequate for severe scale variations and partial visibility that are ubiquitous in multi-person settings. Our RaIoU loss, by contrast, explicitly evaluates spatial alignment through three complementary components: position alignment (via the ${\left(x-x^{*}\right)}^{2}+{\left(y-y^{*}\right)}^{2}$ term), scale consistency (via the log-scale term), and enclosure quality (via the $c^{2}$ denominator). This multi-faceted approach allows the model to learn tighter, more accurate bounding boxes even when substantial portions of the target are occluded.

The dramatic performance improvement of RaIoU validates our hypothesis that modeling occlusion induced spatial uncertainty is essential for reliable posture recognition. In classroom and office settings, where students and workers often sit in close proximity with partial visibility, conventional losses fail to distinguish between acceptable and problematic misalignments. This ablation study not only demonstrates the technical superiority of RaIoU but also reveals the critical importance of domain-specific loss design for vision-based ergonomics applications. The results suggest that generic bounding box regression strategies are insufficient for scenarios with systematic occlusion patterns, and specialized losses that account for the particular spatial constraints of the target domain can yield substantial improvements in both detection accuracy and downstream task performance.

#### 4.5.4. Ablation Analysis of EsBody Key Components

The ablation study on ESBody components reveals the critical importance of context-aware segmentation for robust sitting posture recognition in multi-person environments. As shown in Table 6, the baseline model without ESBody components achieves only 78.62% accuracy, highlighting the severe degradation caused by cross-person interference and occlusion in classroom and office settings.

The incremental addition of ESBody components demonstrates their complementary roles in addressing different aspects of the occlusion problem. The Reno module alone (Settings (a)) improves accuracy by 3.67% to 82.29%, confirming its effectiveness in suppressing boundary-connected components that originate from neighboring individuals. This aligns with the module’s design principle of eliminating non-target regions through connected component filtering, which directly mitigates the “non-target leakage” problem that plagues conventional pipelines.

The most substantial gain comes from incorporating the APF module (Settings (b)), which boosts accuracy by an additional 5.78% to 88.07%. This significant improvement validates APF’s ability to provide high-level semantic understanding of body structure—particularly its lower-body visibility ratio calculation and facial orientation inference. The 5.93 percentage point increase in F1 score (from 83.09% to 89.02%) underscores APF’s critical role in identifying occlusion status and routing samples to appropriate classification branches, which is essential for handling the common scenario of lower-body occlusion in seated environments.

The OpenPose component (Settings (c)) contributes a 7.70 percentage point accuracy improvement over the baseline, though slightly less than APF. This result demonstrates the value of geometric priors and structured skeleton representations, but also reveals that skeleton information alone is insufficient without the semantic context provided by APF. The 1.75 percentage point performance gap between Settings (b) and (c) confirms that semantic understanding of occlusion status (via APF) is more crucial than geometric structure (via OpenPose) for our specific task.

The synergistic effect of all three components in the complete LAViTSPose system is striking: it achieves 94.23% accuracy, a 6.16 percentage point improvement over the best partial configuration (Settings (b)). This non-additive gain (15.61% vs. 5.78% + 7.70% = 13.48%) reveals the critical interdependence of these modules. Reno first cleans the region of interest by removing interference; APF then provides semantic context about occlusion and orientation; and OpenPose converts the refined region into a structured representation that focuses on essential postural features. This cascade of information processing creates a virtuous cycle where each stage benefits from the precision of the previous one.

The ablation study not only validates the design choices of ESBody but also reveals the importance of separating semantic understanding (APF) from geometric representation (OpenPose). In real-world settings where lower-body occlusion is common due to desks and chairs, APF’s ability to detect and route based on occlusion status proves more valuable than pure skeleton information, though both are required for optimal performance. This insight has significant implications for human-centric vision systems operating in constrained environments, suggesting that semantic understanding of occlusion patterns is as important as geometric modeling for robust recognition.

#### 4.5.5. Ablation Analysis of MLiT Key Components

The ablation study on MLiT components reveals the critical importance of specialized local modeling and attention stabilization for robust sitting posture recognition under small-sample training constraints. As shown in Table 7, the baseline model without MLiT components achieves only 80.68% accuracy, highlighting the limitations of standard Transformer architectures in modeling fine-grained postural structures with limited training data.

The SDC module alone (Settings (a)) provides a substantial 3.94 percentage point improvement in accuracy to 84.62%, confirming its effectiveness in enhancing local spatial awareness. This aligns with the module’s design principle of aggregating spatially displaced neighbors before patchification, which injects crucial local inductive bias into the Transformer architecture. In occlusion-prone multi-person settings, this local modeling capability is essential for distinguishing subtle postural variations that global attention mechanisms might overlook, particularly when training data are scarce.

The LT module (Settings (b)) contributes a 0.49 percentage point accuracy improvement, though less pronounced than SDC. This demonstrates the value of learnable temperature scaling in stabilizing attention distributions during training, especially under few-shot conditions. Without this stabilization, attention patterns become erratic and unreliable, particularly when distinguishing between similar postures that differ only in subtle structural details.

These findings have broader implications for vision-based ergonomic assessment systems, suggesting that attention mechanisms in Transformer architectures must be explicitly designed to handle the specific challenges of posture recognition: limited training data, fine-grained structural differences, and occlusion-induced partial visibility. The combination of local structure enhancement of SDC and attention stabilization of LT proves particularly valuable for real-world deployment scenarios where large-scale annotated datasets are impractical to obtain.

### 4.6. Hyperparameter Study

To systematically evaluate the impact of key hyperparameters in the LAViTSPose framework on model performance, we conducted a series of hyperparameter sensitivity analysis experiments.

#### 4.6.1. Hyperparameter Analysis of Range-Aware IoU Loss

This hyperparameter analysis reveals the critical importance of balancing position alignment and scale consistency in the RaIoU loss for multi-person sitting posture recognition. As illustrated in Table 8, the results demonstrate that equal weighting of the position term ($\lambda_{\mathrm{pos}}$) and scale term ($\lambda_{\mathrm{size}}$) at 0.5 each achieves optimal performance across all metrics, with a significant 4.0–4.5 percentage point improvement over imbalanced configurations.

The performance gap between the balanced configuration (0.5/0.5) and the alternatives (0.7/0.3 and 0.3/0.7) highlights the unique challenges of sitting posture recognition in classroom and office environments. When $\lambda_{\mathrm{pos}}$ is too high (0.7/0.3), the model prioritizes center-point alignment but becomes insensitive to aspect-ratio mismatches, which are common when students sit at different distances from the camera or adopt various postures (e.g., upright vs. slouched). Conversely, when $\lambda_{\mathrm{size}}$ dominates (0.3/0.7), the model becomes overly sensitive to scale variations but fails to adequately suppress “non-target leakage” from neighboring individuals, leading to contaminated regions of interest.

The optimal 0.5/0.5 balance reflects the specific requirements of our application domain: in crowded indoor settings where desk/peer occlusions are pervasive, precise center-point positioning is equally important as accurate scale estimation. For instance, when a student is partially occluded by a desk or neighbor, the model must simultaneously localize the visible portion correctly (position term) while maintaining appropriate bounding box dimensions (scale term) to avoid including non-target regions.

This finding helps explain why our end-to-end accuracy remains at 94.23% under the best settings. The precise bounding boxes generated by YOLOR with balanced RaIoU loss provide clean input for the ESBody segmentation module, which in turn enables the MLiT classifier to operate on high-quality structural features without interference. This inter-stage coherence is fundamental to the framework’s robustness in real-world classroom environments where individuals frequently sit in close proximity with partial visibility.

#### 4.6.2. Hyperparameter Analysis of NMS Threshold

The NMS threshold $\tau_{nms}$, in conjunction with the objectness threshold $\tau_{obj}$, represents a critical hyperparameter pair that governs the trade-off between detection completeness and redundancy suppression in crowded scenes. As shown in Table 9, the configuration $\tau_{obj}=0.50$, $\tau_{nms}=0.50$ achieves optimal end-to-end performance, yielding 94.23% accuracy, 92.02% precision, and 92.34% recall. This balanced setting effectively retains partially visible individuals under desk or peer occlusion while suppressing duplicate detections that would otherwise introduce cross-person interference.

In contrast, the aggressive setting $\tau_{obj}=0.70$, $\tau_{nms}=0.30$ over-suppresses overlapping detections, significantly reducing recall to 88.77% and degrading overall accuracy to 88.31%. This loss is particularly detrimental in classroom scenarios where students seated side-by-side often share boundary regions; valid detections are mistakenly pruned, leading to missed subjects. On the other hand, the permissive setting $\tau_{obj}=0.30$, $\tau_{nms}=0.70$ allows redundant boxes to survive NMS, resulting in multiple detections for the same person. Although recall remains relatively high (91.72%), the contaminated regions of interest introduce non-target body parts into ESBody, causing cross-person leakage and reducing accuracy to 91.18%.

These results underscore that domain-specific tuning of detection thresholds is essential for sitting posture recognition. The optimal $\left(\tau_{obj},\tau_{nms}\right)=\left(0.50,0.50\right)$ ensures that ESBody receives clean, single-person ROIs, which in turn enables the MLiT classifier to operate on high-fidelity structural inputs. This precise control over detection quality is a key enabler of our framework’s robustness in real-world, multi-person indoor environments.

#### 4.6.3. Hyperparameter Analysis of Reno

As shown in Table 10, this experimental analysis demonstrates the critical impact of Reno’s hyperparameters on multi-person sitting posture recognition performance. The lower foreground threshold $\tau_{m}=0.3$ proves more effective than 0.5 at preserving weak foreground signals in classroom scenarios, particularly for students wearing dark clothing or under complex lighting conditions. These boundary pixels frequently correspond to partially visible shoulder or head contours, which serve as essential discriminative features for distinguishing subtle posture variations like “Upright” versus “Leaning-forward.” A higher $\tau_{m}$ value (0.5) prematurely discards these critical edge pixels, resulting in incomplete body masks that degrade classification accuracy. Meanwhile, the length threshold $\tau_{\ell }=1.2$ (corresponding to T=1.2(m+n)) optimally removes approximately 10–15% of boundary-connected components, which typically belong to neighboring students’ limbs, thereby effectively preventing cross-person feature contamination.

In contrast, the higher $\tau_{\ell }=1.5$ setting allows excessive boundary-connected components to remain, resulting in 93.14% accuracy, 1.1% lower than the optimal configuration. While this alternative achieves a slightly better F1 score (92.52% vs 92.18%), the more significant drop in accuracy demonstrates that precision is more critical than recall for sitting posture recognition. Contaminated regions directly introduce false structural features that mislead the classifier, particularly for subtle posture differences. This parameter sensitivity validates the necessity of Reno’s design for classroom-specific spatial patterns: by precisely tuning these thresholds, the system can effectively suppress cross-person interference while preserving critical body parts, ultimately achieving 94.23% accuracy through the provision of high-quality inputs to the subsequent classification stage.

#### 4.6.4. Hyperparameter Analysis of APF

As shown in Table 11, the hyperparameter settings of the APF module have a decisive impact on the overall performance of the LAViTSPose framework. The third parameter configuration ($r_{LB}^{thr}=0.60$, $r_{L}^{thr}=0.67$, $r_{R}^{thr}=0.75$) achieves the highest accuracy of 94.23%, which is 4.9% to 5.8% higher than other configurations. This significant difference stems from the direct impact of threshold settings on the routing decision accuracy of the APF module: the lower limb visibility threshold $r_{LB}^{thr}=0.60$ appropriately identifies partially occluded scenarios (routing samples to the HB branch when visibility falls below this value), avoiding both the excessive routing caused by the first parameter set ($r_{LB}^{thr}=0.1$) where most samples incorrectly enter the HB branch, and the critical occlusion scenarios being overlooked by the second parameter set ($r_{LB}^{thr}=1.7$). The facial orientation thresholds $r_{L}^{thr}=0.67$ and $r_{R}^{thr}=0.75$ enable sensitive recognition of slight facial deviations through precise comparison of activation intensities in left and right facial regions, ensuring the classifier can select the most appropriate processing path based on actual visible information.

The optimal parameter configuration not only improves overall accuracy but also achieves a balance between precision (92.02%) and recall (92.34%), indicating that the APF module can effectively assign samples to the correct processing branch under this setting. Excessively low thresholds (first configuration) result in both low precision and recall, suggesting the system cannot distinguish between valid and invalid features; while excessively high thresholds (second configuration) slightly improve precision but decrease recall, indicating the system misses many occlusion scenarios that should be recognized. This optimization of the APF module validates its design principle: by analyzing the 24-part probability maps output by BodyPix to infer lower limb visibility and facial orientation, it provides reliable routing decisions for subsequent classification. This mechanism enables LAViTSPose to adapt to complex occlusion patterns in multi-person sitting posture scenarios, delivering high-quality structural features to the MLiT classifier and ultimately achieving 94.23% accuracy.

#### 4.6.5. Hyperparameter Analysis of SDC

As shown in Table 12, both the neighborhood Δ and the displacement δ materially affect performance. The configuration with an 8-connected neighborhood (N8) and a 1-pixel displacement (δ=1) attains the best results of 94.23% accuracy and 92.18% F1—exceeding other configurations by 2.0% to 5.0% in accuracy and 0.5% to 3.0% in F1. This suggests that richer spatial context from diagonal neighbors helps capture fine-grained structural cues (e.g., shoulder alignment, torso tilt) that differentiate subtle postures such as Upright vs. Leaning-forward.

Viewing the two factors factorially, the main effect of enlarging the neighborhood from N4 to N8 is consistently positive (F1: +3.0 pp at δ=1 and +0.7 pp at δ=2). The main effect of increasing displacement depends on Δ: with N4, moving from δ=1 to δ=2 improves F1 by +1.8 pp (more context is beneficial); with N8, the same change slightly reduces F1 by −0.5 pp. This interaction indicates that when the neighborhood is already rich (N8), larger shifts start to introduce background interference or misalignment in crowded scenes, which mildly lowers both precision (from 92.02% to 91.78%) and recall (from 92.34% to 91.60%).

Overall, (N8, δ=1) offers a balanced trade-off: it expands the local receptive field without compromising feature fidelity. Under occlusion and limited data, this setting injects a useful inductive bias into the lightweight ViT backbone and yields strong posture recognition performance.

### 4.7. Few-Shot Learning Performance

The few-shot learning experiment demonstrates LAViTSPose’s superior performance when trained with only 50% of the labeled data, highlighting its remarkable data efficiency and robustness under annotation scarcity. As shown in Table 13, our framework achieves 87.62% accuracy, outperforming the second-best CaiT by 4.45% and standard ViT by 4.89%. This significant improvement is particularly noteworthy in the context of sitting posture recognition, where obtaining high-quality annotations is challenging due to privacy concerns, complex occlusion patterns, and the labor-intensive nature of fine-grained posture labeling.

The performance gap becomes even more pronounced when examining the F1 score, where LAViTSPose achieves 87.94% compared to 83.82% for CaiT. This indicates that our framework maintains a better balance between precision and recall under data constraints, which is crucial for reliable posture classification in classroom settings where both false positives and false negatives can lead to incorrect ergonomic assessments.

This experiment validates our claim that for specialized domains like sitting posture recognition, where large-scale annotations are impractical, a framework with domain-specific architectural innovations is more effective than simply scaling generic models. LAViTSPose’s ability to achieve 87.62% accuracy with half the labeled data would translate to substantial cost savings in real-world deployment scenarios, making it a practical solution for educational institutions and workplaces that need ergonomic monitoring but lack resources for extensive data labeling.

### 4.8. Visualization Analysis

**Figure 6:**
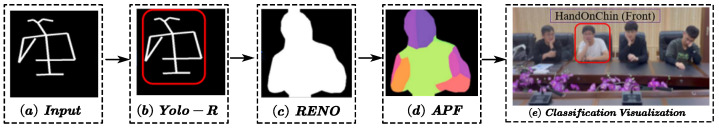
Visual example of the LAViTSPose pipeline, including pixel-level skeleton segmentation and final posture classification. The red bounding box indicates the detected target person, and the different colors in the segmentation map represent different body parts.

To better understand the model’s decision-making process and the role of the segmentation-classification cascade, we visualize the complete pipeline on real-world test images. As shown in Figure 6, our framework successfully identifies complex postures such as HandOnChin, even under partial occlusion and in cluttered multi-person scenes.

These results highlight the synergy between the detection, segmentation, and classification modules. The YOLOR detector first isolates individuals with precise bounding boxes, the ESBody segmenter (with Reno and APF) then provides pixel-level body parsing that suppresses cross-person interference while maintaining anatomical structure, and the MLiT classifier leverages this structured representation for accurate posture recognition. This cascaded approach enables better generalization and robustness than traditional end-to-end pipelines.

In summary, the component-wise evaluation confirms the effectiveness of our detection–segmentation–classification cascade design. YOLOR ensures efficient and accurate person localization, ESBody provides fine-grained semantic understanding while handling occlusion, and the MLiT-based classifier leverages this structured representation for reliable posture recognition. Together, these modules form a cohesive pipeline optimized for real-world sitting posture analysis under occlusion, overlap, and limited data conditions.

## 5. Conclusions

This paper introduces LAViTSPose, a lightweight augmented framework that addresses the critical challenges of multi-person sitting posture recognition in real-world classroom and office environments, where pervasive occlusions and proximity-induced interference severely degrade conventional approaches. Through our cascaded design—integrating occlusion-robust YOLOR detection, interference-suppressing ESBody segmentation, and local-structure-enhanced MLiT classification—we achieve state-of-the-art 94.2% accuracy while maintaining real-time inference capabilities, demonstrating that targeted architectural innovations rather than brute-force scaling are essential for reliable ergonomics assessment in resource-constrained settings. The framework’s effectiveness stems from its explicit error mitigation at each processing stage: RaIoU loss stabilizes bounding box regression under partial visibility; ESBody’s Reno module suppresses cross-person interference while APF provides semantic routing without requiring additional training; and MLiT’s SDC and LT mechanisms enhance local modeling with limited annotations, enabling robust performance with 54.2M parameters—significantly fewer than competing ViT-based approaches. From an information-theoretic perspective, the framework implicitly addresses entropy by minimizing uncertainty across each stage. This work establishes a practical foundation for scalable ergonomic monitoring systems that can operate effectively under real-world constraints, with potential applications in education, workplace wellness, and public health. Future research will explore end-to-end optimization of the pipeline, extension to temporal analysis for continuous monitoring, and integration with complementary sensing modalities to further improve robustness in complex environments.

## Figures and Tables

**Table 1:** Comparison of different network architectures in the sitting posture recognition task. Best performance in each setting is highlighted in bold.

Network	Acc (%)	Prec (%)	Rec (%)	F1 (%)	Flops (G)	Params (M)	Time (ms)
VGG16 [43]	89.42	87.81	88.04	87.92	15.51	138.4	27.33
VGG19 [43]	83.92	81.44	87.57	84.39	19.63	143.7	29.63
MobileNet [44]	87.76	83.21	88.15	85.61	0.33	2.23	24.47
ResNet18 [45]	88.34	84.63	91.31	87.84	1.87	11.7	29.58
ResNet101 [46]	91.38	89.81	92.43	91.10	7.88	46.7	46.32
ViT-Transformer [34]	92.82	89.37	94.58	91.90	16.86	85.7	36.13
PiT [47]	90.76	86.64	91.85	89.17	13.27	111.0	38.88
CaiT [48]	90.81	88.05	89.47	88.75	14.07	121.3	41.22
**LAViTSPose (Ours)**	**94.23**	**92.02**	**92.34**	**92.18**	10.73	54.2	34.17

**Table 2:** Ablation study on object-detection modules. CBP denotes the learnable normalization layer; SiLU denotes the Sigmoid Linear Unit activation; PAN-FPN denotes the Path Aggregation + Feature Pyramid Network for multi-scale feature fusion. Best performance in each setting is highlighted in bold.

Settings	Object Detection			Evaluation Metrics			
	CBP	SiLU	PAN-FPN	Acc (%)	Prec (%)	Rec (%)	F1 (%)
Baseline				81.12	82.31	81.72	82.01
Settings (a)	✓			83.24	83.62	83.03	83.32
Settings (b)	✓	✓		85.38	85.88	85.69	85.78
Settings (c)			✓	86.33	87.00	86.81	86.90
**LAViTSPose (Ours)**	✓	✓	✓	**94.23**	**92.02**	**92.34**	**92.18**

**Table 3:** Detector sweep under a fixed pipeline: ESBody segmentation and MLiT classifier are trained once using our YOLOR-based detector and then frozen across all detector variants. Metrics are end-to-end classification results. Best performance in each setting is highlighted in bold.

Method	Acc (%)	Prec (%)	Rec (%)	F1 (%)
YOLO-v3	83.31	83.38	83.73	83.55
YOLO-v4	85.02	85.41	85.02	85.21
YOLO-v5s	88.58	89.17	88.83	89.00
YOLO-v7	91.21	92.00	91.51	91.75
YOLO-v9	92.42	91.71	91.83	91.77
YOLO-v11	94.03	**92.20**	92.14	92.17
YOLOR + Ralou (ours)	**94.23**	92.02	**92.34**	**92.18**

**Table 4:** Person detection performance (COCO-style) of YOLO variants, including the original YOLOR [25] and our customized YOLOR-based detector (with SiLU, CBP, PAN-FPN, anchor re-estimation, and RaIoU loss). Best performance in each setting is highlighted in bold.

Method	mAP@0.5 (%)	Speed (FPS)
YOLO-v3	89.82	22.17
YOLO-v4	91.33	39.42
YOLO-v5s	91.91	60.35
YOLOR	91.24	57.81
YOLO-v7	88.18	51.14
YOLO-v9	90.20	57.46
YOLO-v11	92.60	**65.37**
YOLOR + Ralou (ours)	**93.12**	60.15

**Table 5:** Ablation study on bounding box regression losses under identical detector architecture and training protocol. Only the regression loss term is varied. RaIoU achieves the best detection and downstream classification performance. Best results are highlighted in bold.

Loss Function	Evaluation Metrics			
	Acc (%)	Prec (%)	Rec (%)	F1 (%)
$L_{IoU}$ [23]	88.42	88.62	88.33	88.47
$L_{DIoU}$ [24]	89.44	90.29	89.57	89.93
$L_{CIoU}$ [24]	90.21	90.71	90.33	90.52
$L_{GIoU}$ [23]	89.88	90.18	89.62	89.90
$L_{RIoU}$ [25]	90.76	91.44	91.16	91.30
**$L_{RaIoU}$ (Ours)**	**94.23**	**92.02**	**92.34**	**92.18**

**Table 6:** Ablation study on ESBody components. Best performance in each setting is highlighted in bold.

Settings	ESBody			Evaluation Metrics			
	Reno	APF	OpenPose	Acc (%)	Prec (%)	Rec (%)	F1 (%)
Baseline				78.62	79.20	78.88	79.04
Settings (a)	✓			82.29	83.42	82.76	83.09
Settings (b)	✓	✓		88.07	89.32	88.73	89.02
Settings (c)	✓		✓	86.32	87.31	87.44	87.37
**LAViTSPose (Ours)**	✓	✓	✓	**94.23**	**92.02**	**92.34**	**92.18**

**Table 7:** Ablation study on MLiT components. SDC stands for Spatial Displacement Contact module, and LT refers to learnable temperature mechanism. Best performance in each setting is highlighted in bold.

Settings	MLiT		Evaluation Metrics			
	SDC	LT	Acc (%)	Prec (%)	Rec (%)	F1 (%)
Baseline			80.68	81.13	80.91	81.02
Settings (a)	✓		84.62	85.70	85.33	85.51
Settings (b)		✓	81.17	83.13	82.28	82.70
**LAViTSPose (Ours)**	✓	✓	**94.23**	**92.02**	**92.34**	**92.18**

**Table 8:** Hyperparameter analysis of Range-aware IoU loss. Best performance in each setting is highlighted in bold.

Hyperparameter	Evaluation Metrics			
	**Acc (%)**	**Prec (%)**	**Rec (%)**	**F1 (%)**
**$\lambda_{\mathrm{pos}}=0.7$ $\lambda_{\mathrm{size}}=0.3$**	89.71	90.16	89.93	90.04
**$\lambda_{\mathrm{pos}}=0.5$ $\lambda_{\mathrm{size}}=0.5$**	**94.23**	**92.02**	**92.34**	**92.18**
**$\lambda_{\mathrm{pos}}=0.3$ $\lambda_{\mathrm{size}}=0.7$**	90.22	90.54	90.21	90.37

**Table 9:** Hyperparameter sensitivity analysis of Non-Maximum Suppression (NMS) threshold in YOLOR. Best performance in each setting is highlighted in bold.

Hyperparameter	Evaluation Metrics			
	Acc (%)	Prec (%)	Rec (%)	F1 (%)
**$\tau_{obj}=0.70$ $\tau_{nms}=0.30$**	88.31	89.14	88.77	88.95
$\tau_{obj}=0.50$ $\tau_{nms}=0.50$	**94.23**	**92.02**	**92.34**	**92.18**
$\tau_{obj}=0.30$ $\tau_{nms}=0.70$	91.18	92.01	91.72	91.86

**Table 10:** Reno hyperparameters $\tau_{m}$ (foreground threshold) and $\tau_{\ell }$. Best performance in each setting is highlighted in bold.

Hyperparameter	Evaluation Metrics			
	Acc (%)	Prec (%)	Rec (%)	F1 (%)
$\tau_{m}=0.3$ $\tau_{l}=1.2$	**94.23**	92.02	92.34	92.18
$\tau_{m}=0.5$ $\tau_{l}=1.5$	93.14	**92.35**	**92.70**	**92.52**

**Table 11:** APF thresholds: lower-body visibility $r_{\mathrm{LB}}^{\mathrm{thr}}$ and asymmetric head-orientation ratios $\left(r_{L}^{\mathrm{thr}},r_{R}^{\mathrm{thr}}\right)$. Best performance in each setting is highlighted in bold.

Hyperparameter	Evaluation Metrics			
	Acc (%)	Prec (%)	Rec (%)	F1 (%)
$r_{LB}^{thr}=0.1$ $r_{L}^{thr}=0.15$ $r_{R}^{thr}=0.2$	88.44	89.03	89.22	89.12
$r_{LB}^{thr}=1.7$ $r_{L}^{thr}=1.5$ $r_{R}^{thr}=1.3$	89.33	90.11	89.45	89.78
$r_{LB}^{thr}=0.60$ $r_{L}^{thr}=0.67$ $r_{R}^{thr}=0.75$	**94.23**	**92.02**	**92.34**	**92.18**

**Table 12:** A 2 × 2 factorial study of SDC with neighborhood Δ∈{N4,N8} (4- vs. 8-connected) and pixel displacement δ∈{1,2} (pixels). Best value per metric is in bold.

Δ	*δ*	Acc (%)	Prec (%)	Rec (%)	F1 (%)
N4	1	89.22	89.37	89.04	89.20
N4	2	90.14	91.26	90.81	91.03
N8	1	**94.23**	**92.02**	**92.34**	**92.18**
N8	2	92.20	91.78	91.60	91.69

**Table 13:** Few-shot learning performance comparison with 50% labeled training data. Best performance in each setting is highlighted in bold.

Model	Evaluation Metrics (50% Lable)			
	Acc (%)	Prec (%)	Rec (%)	F1 (%)
ResNet101	80.10	81.22	80.93	81.07
ViT	82.73	83.08	82.73	82.90
PiT	81.42	81.90	81.56	81.73
CaiT	83.17	84.03	83.61	83.82
**LAViTSPose (Ours)**	**87.62**	**88.08**	**87.80**	**87.94**

## Data Availability

The original contributions presented in the study are included in the article; further inquiries can be directed to the corresponding author.
